# Screening of Natural Products and Approved Oncology Drug Libraries for Activity against *Clostridioides difficile*

**DOI:** 10.1038/s41598-020-63029-0

**Published:** 2020-04-06

**Authors:** Rusha Pal, Mohamed N. Seleem

**Affiliations:** 1 Department of Comparative Pathobiology, College of Veterinary Medicine, Purdue University, West Lafayette, IN 47907 USA; 2 Purdue Institute of Inflammation, Immunology, and Infectious Disease, West Lafayette, IN 47907 USA

**Keywords:** Drug discovery, Microbiology, Infectious diseases

## Abstract

*Clostridioides difficile* is the most common cause of healthcare-associated diarrhea. Infection of the gastrointestinal tract with this Gram-positive, obligate anaerobe can lead to potentially life-threatening conditions in the antibiotic-treated populace. New therapeutics are urgently needed to treat this infection and prevent its recurrence. Here, we screened two libraries from the National Cancer Institute, namely, the natural product set III library (117 compounds) and the approved oncology drugs set V library (114 compounds), against *C. difficile*. In the two libraries screened, 17 compounds from the natural product set III library and 7 compounds from the approved oncology drugs set V library were found to exhibit anticlostridial activity. The most potent FDA-approved drugs (mitomycin C and mithramycin A) and a promising natural product (aureomycin) were further screened against 20 clinical isolates of *C. difficile*. The anticancer drugs, mitomycin C (MIC_50_ = 0.25 μg/ml) and mithramycin A (MIC_50_ = 0.015 μg/ml), and the naturally derived tetracycline derivative, aureomycin (MIC_50_ = 0.06 μg/ml), exhibited potent activity against *C. difficile* strains. Mithramycin A and aureomycin were further found to inhibit toxin production by this pathogen. Given their efficacy, these compounds can provide a quick supplement to current treatment to address the unmet needs in treating *C. difficile* infection and preventing its recurrence.

## Introduction

*Clostridioides difficile* is the leading cause of health care-associated diarrhea and mortality in the United States^1–4^. The Centers for Disease Control and Prevention (CDC) classified *C. difficile* as an urgent threat due to the immense suffering and death of thousands of patients each year. As per the 2017 estimates, the bacterium accounted for 223,900 cases with 12, 800 deaths and an attributable health care cost of $ 1 billion^5^. The characteristic manifestation of *C. difficile*-associated diarrhea (CDAD) ranges from mild diarrhea to pseudomembranous colitis, and toxic megacolon, and 1 out of every 11 patients aged 65 years or older dies within 30 days of diagnosis^6^.

Antibiotic therapy remains a pivotal risk factor, as it aids the growth of *C. difficile* in the colon^7^. In its unperturbed state, the indigenous microflora of the colon serve as a host defense mechanism by providing resistance against colonization of the pathogen^8^. The use of antibiotics disrupts the host microflora, rendering individuals susceptible to *C. difficile* infection (CDI). In the absence of these gut microflora, following oral ingestion, the dormant *C. difficile* spores germinate, colonize the vacant nutrient niche in the gut, and release the enterotoxin TcdA and the cytotoxin TcdB^9,10^. These toxins A and B constitute the major virulence factors of the pathogen that damage the colonic epithelium triggering inflammatory responses that cause the range of symptoms associated with CDI^10,11^. Paradoxically, the standard treatment options for CDI includes oral administration of the antibiotics vancomycin or fidaxomicin^12^. While the use of these antibiotics can alleviate the symptoms associated with CDI, such a treatment regimen can further eliminate commensal bacteria and fails to prevent *C. difficile* reinfection^13^. Hence, recurrence of infection is a common scenario, with initial recurrence rates approaching 30% and secondary recurrences being even more frequent^10^. Novel therapeutic approaches, such as fecal microbiota transplantation (FMT), are being incorporated in the treatment repertoire to address recalcitrant CDI. Primarily considered a last-ditch effort for combating recurrence, the use of this biotherapeutic approach has demonstrated efficacy in treating the microbial dysbiosis that leads to CDI and its recurrence^14,15^. However, the FMT process recently came under FDA (U.S. Food and Drug Administration) scrutiny following the development of invasive infections caused by extended-spectrum-beta-lactamase (ESBL)-producing *Escherichia coli*, which led to the death of one individual following FMT treatment. The limitations in the current treatment options for CDI create an unmet medical need for novel antibacterial agents that can target the pathogen and prevent the recurrence of infection.

Despite recent technological advancements, the development of any novel drug costs an average of $2 - $3 billion and takes up to 13–15 years^16,17^. Drug repurposing, also termed as “drug repositioning, reprofiling, or retasking”, is an advantageous strategy for identifying novel applications for previously approved or investigational drugs^18,19^. A deluge of research indicates that existing medications can be used to treat diseases other than the conditions for which the drugs acquired regulatory approval^20–25^. This is evident from the successful repurposing of sildenafil citrate (an antihypertensive drug that got approved for the treatment of erectile dysfunction), celecoxib (an anti-inflammatory drug being used to treat familial adenomatous polyps), and zidovudine (an anticancer drug repurposed to be the first anti-HIV drug) to name a few^19,20^. The current study involved screening two libraries from the National Cancer Institute (NCI), namely, the natural products set III library (117 compounds) and the approved oncology drugs set V library (114 compounds), with an aim of identifying new drugs/ compounds that can inhibit *C. difficile*.

## Results and Discussion

*C. difficile* has emerged as a bacterium that is challenging to eradicate and is the most common cause of health care-related infection in the United States. The current clinical practice guidelines for CDI include the use of oral vancomycin and fidaxomicin as first-line agents for both nonsevere and severe episodes of infection^26^. Hitherto a drug of choice for CDI treatment, metronidazole is no longer recommended following a 2018 update in the clinical practice guidelines due to potential risks of neurotoxicity^27^. Vancomycin, one of the two prescribed drugs, is relatively successful in the treatment of the initial episode, but recurrence of infection occurs in 20–30% of the patients treated with this therapy. A novel macrocyclic antibiotic, fidaxomicin, was approved by the FDA in 2011 and has an enhanced post-antibiotic effect, with a lesser impact on the gut microbiome of the host compared to that of vancomycin. However, the disadvantages associated with fidaxomicin include its prohibitive cost, lack of efficacy in patients infected with the NAP1/PCR ribotype 027 strains, and observed resistance in clinical settings^28,29^. Alternative strategies for treating patients with multiple recrudescence, such as FMT, lack standardization, and the long-term ramifications of such procedures remain unknown^2^. The looming threat posed by the pathogen and the lack of an effective treatment regimen necessitates novel scaffolds for the treatment of CDI. Translation of the current knowledge and technological advancements to a novel therapeutic is shackled by multifold challenges, including high costs, increased time, and risks of failure, thus making it a less preferable choice for investors. Drug repurposing, which involves identifying novel uses for drugs^30–33^ that have already been approved or are in early stages of clinical trials, can be a lucrative alternative strategy, as it offers a reduced time frame for development, less costly investment, and lower risks of failure^18,34^.Thus, with this approach in mind, we screened two NCI libraries in an attempt to identify novel scaffolds exhibiting anti-clostridial activity.

### Screening assay and broth microdilution assay

The approved oncology drugs set V library (consisting of 114 FDA-approved drugs) and the natural products set III library (consisting of 117 compounds including seven FDA-approved natural products) were screened at a concentration of 16 μM for anti-*C. difficile* properties. In the initial screening assay, 7 hits were identified from the approved oncology drugs set V library, and 17 hits were identified from the natural products set III library. The initially identified hits were selected, and the minimum inhibitory concentrations (MIC) of the compounds were confirmed, starting at a concentration of 32 μM. The MIC values for these compounds were found to range between ≤ 0.25 μM and 32 μM (Tables 1 and 2; Supplementary Tables S2 and S3).

**Table 1:** Initial screening data for the approved oncology drugs set V library against *C. difficile* ATCC BAA 1807.

	Compound name	MIC (μM)
1	Ponatinib	16
2	Regorafenib	8
3	Sorafenib	8
4	Mitomycin C	0.5
5	Tamoxifen citrate	16
6	Actinomycin D	32
7	Mithramycin A/ Plicamycin	≤0.25

**Table 2:** Initial screening data for the natural product set III library against *C. difficile* ATCC BAA 1807.

	Compound name	MIC (μM)
1	Aureomycin	0.5
2	Norlobaric acid	32
3	Actinomycin D	32
4	Valinomycin	32
5	Pomiferin	32
6	Aristolochin	32
7	Mangostin	16
8	Siomycin A	≤0.25
9	Lonchocarpic acid	32
10	Tetrocarcin A, sodium salt	0.5
11	Michellamine B	16
12	Rifamycin	≤0.25
13	Nigericin	≤0.25
14	Antibiotic X-536A	1
15	Chaetochromin	0.5
16	Levomycin	≤0.25
17	Gangetin	8

Of the approved oncology drugs, the most potent compounds included mitomycin C (0.5μM) and plicamycin/ mithramycin A (≤0.25 μM) (Tables 1 and 2; Supplementary Tables S2 And S3). The natural product library had several compounds with potent anticlostridial activity which included aureomycin (0.5μM), siomycin A (≤0.25 μM), tetrocarcin A (0.5 μM), rifamycin (≤0.25 μM), nigericin (≤0.25 μM), antibiotic X-536A (1 μM), chaetochromin (0.5 μM), and levomycin (≤0.25 μM) (Tables 1 and 2; Supplementary Tables S2 and S3). As *C. difficile* remains in the lumen of the colon, an ideal antibiotic would be the one that is retained in the colon with potent anti-toxin and anti-clostridial activities^35^. Rifaximin, a rifamycin derivative that is poorly absorbed from the intestine, has already been found to exhibit potent activity against *C. difficile* isolates and is considered to be a follow-up therapy for treating CDI recurrence^36^; hence, it was not investigated further. Of the other potent natural products/antibiotics, aureomycin, is known to be poorly absorbed from the intestine^37^. Furthermore, aureomycin belongs to the tetracycline class of antibiotics, which is associated with a lower risk of primary CDI and acts by inhibiting the binding of the aminoacyl tRNA to the mRNA ribosome complex^38,39^. Hence, because of its promising pharmacological properties, the aureomycin scaffold was selected for further studies, along with the two FDA-approved oncology drugs, mitomycin C and mithramycin A.

### Activity of the selected drugs against a panel of *C. difficile* strains

Mitomycin C is currently an FDA-approved chemotherapeutic agent for the treatment of bladder, gastric, and pancreatic cancer treatment. An antineoplastic antibiotic and a potent DNA alkylating agent, mitomycin C, isolated from *Streptomyces caespitosus* and other *Streptomyces* sp.^40^, has previously been shown to have antibacterial activity against planktonic, biofilm, and metabolically dormant persister cells of *E. coli*, *Staphylococcus aureus*, and *Pseudomonas aeruginosa*, with MIC_50_ values ranging from 0.2–15 μg/ml^41^. Furthermore, this aziridine-containing compound has also been reported to be active against stationary phase and persister cells of *Borrelia burgdorferi* with an MIC value of 0.2 μg/ml^41,42^. Here, we report the anti-*C. difficile* property of mitomycin C with an MIC_50_ value of 0.25 μg/ml when tested against a panel of 20 clinically relevant *C. difficile* strains (Table 3).

**Table 3:** Minimum inhibitory concentration (MIC) values of mitomycin C, mithramycin A, and aureomycin and control antibiotic vancomycin against clinical and hypervirulent strains of *C. difficile*.

*C. difficile* strains	NR number	MIC (μg/ml)			
		Mitomycin C	Mithramycin A	Aureomycin	Vancomycin
P2	32883	0.25	0.015	0.03	0.25
P6	32886	0.5	0.015	0.125	0.5
P7	32887	0.5	0.03	0.125	0.5
P8	32888	0.5	0.015	0.125	0.25
P9	32889	0.25	0.015	0.06	1
P19	32895	0.25	0.03	0.06	1
Isolate 1	13427	1	0.03	0.125	1
Isolate 2	13428	0.125	0.015	0.03	0.25
Isolate 4	13430	0.125	0.03	0.06	0.25
Isolate 6	13432	0.5	0.06	0.06	0.5
Isolate 9	13435	0.5	0.015	0.06	0.5
Isolate 10	13436	0.5	0.015	0.06	0.25
Isolate 20100502	49277	0.5	0.03	0.06	0.25
Isolate 20100207	49278	0.25	0.015	0.03	0.25
Isolate 20100211	49279	0.25	0.015	0.03	0.25
Isolate 20120016	49282	0.25	0.015	0.03	0.25
Isolate 20110999	49286	0.25	0.015	0.125	0.25
Isolate 20110870	49288	0.25	0.015	0.06	0.25
Isolate 20120187	49290	0.25	0.015	0.06	0.25
ATCC BAA 1870		0.25	0.015	0.125	1
MIC_50_		0.25	0.015	0.06	0.25

Mithramycin A, an anthracycline antibiotic and an aureolic acid, isolated from the bacterium *Streptomyces argillaceus*^43^, is known to exhibit antibacterial properties against Gram-positive bacteria^44^. Mithramycin A was found to exhibit potent activity against *C. difficile* strains with an MIC_50_ value of 0.015 μg/ml (Table 3). A factor that needs to be considered when choosing drugs for the treatment of CDI is the ability of the drug to be retained in the colon. This point is best exemplified by the reduced activity of metronidazole in comparison to vancomycin in CDI treatment, despite metronidazole exhibiting more potent activity in vitro^45,46^. The poorer efficacy of metronidazole has been attributed to its high absorption from the upper GI tract, with very low levels reaching the colon. In contrast, vancomycin is not absorbed and can thus attain high concentrations in the colon^45^. Mithramycin A is administered via the intravenous route, as it is poorly absorbed when given orally^47^. This pharmacokinetic property of mitomycin C and mithramycin A, along with their anti*-C. difficile* trait, can enhance treatment outcomes for CDI.

Aureomycin, a member of the tetracycline class and isolated from *Streptomyces aureofaciens*, is known for its antiprotozoal and antibacterial activities^48^. The MIC_50_ value of aureomycin against *C. difficile* was found to be 0.06 μg/ml (Table 3). The burgeoning use of antibiotics pose an increased risk of CDI because antibiotics disrupt the host microbiota, thus facilitating *C. difficile* proliferation in the gut. However, tetracyclines are a group of antibiotics that have been associated with a decreased risk of CDI development^38^. The use of aureomycin to treat CDI, can therefore resolve the conundrum associated with the use of traditional antibiotics (vancomycin and fidaxomicin) and the recurrence of infection. Aureomycin, a chlortetracycline, is also poorly absorbed from the GI tract, and high concentrations of the drug can be achieved in the intestine^37^. Hence, the potency of aureomycin, along with its poor absorption, makes it an interesting scaffold that can be pursued for the development of new drugs in the near future.

### Toxin inhibition assay

Although an interplay between several virulence factors helps to colonize the pathogen in the intestine, the key determinant of *C. difficile* virulence is its ability to produce toxins^49^. The pathogenic strains of *C. difficile* express two large, homologous, glycosylating toxins, TcdA and TcdB, and a binary toxin called CDT^50^. These toxins inactivate the small Rho-family GTPases, leading to the loss of the cell integrity and eventual cell death, thereby injuring the colonic epithelium^51^. Furthermore, these toxins stimulate the release of the proinflammatory cytokines and neutrophil chemoattractants which culminates in an acute inflammatory response that plays a central role in *C. difficile*-associated diarrhea and colitis^52^. Of the first-line therapeutic agents used to treat CDI, only fidaxomicin can suppress expression of the *tcdA* and *tcdB* genes, resulting in the inhibition of toxin production^53^. However, the dramatic rise in the treatment failure and the relapse rate associated with the current antibiotics both emphasize the need for alternative therapeutics that can neutralize toxin function.

**Figure 1:**
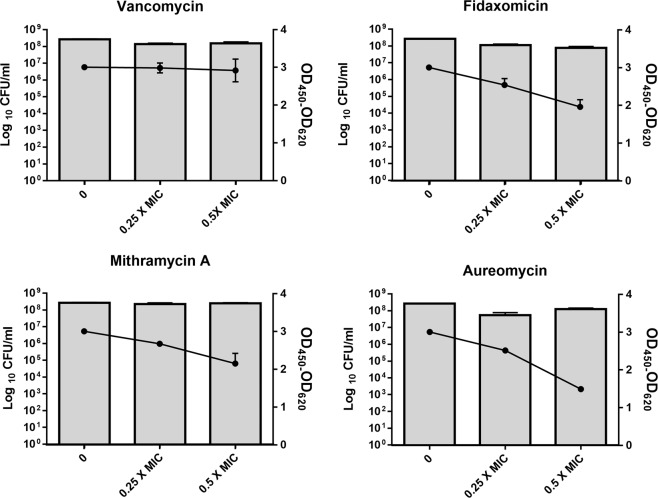
Toxin inhibition activity of mithramycin A, aureomycin and control anticlostridial drugs (vancomycin and fidaxomicin) against *C. difficile*. Drugs at 0.25X and 0.5X MIC were incubated with a hypervirulent, toxigenic strain of *C. difficile* (strain ATCC BAA-1870) for 12 hours. Viable cells (log_10_ CFU/ml, bars) were counted and the toxin content in each supernatant (OD_450_ − OD_620_, lines) was assessed using enzyme-linked immunosorbent assay (ELISA). Data are mean and standard deviation for each antibiotic treatment done in triplicate.

Mithramycin A and aureomycin were further selected for determining the inhibitory action of these compounds on toxin production by *C. difficile* ATCC BAA 1870. Aureolic acids, which include mithramycin A, are known to interact with the high GC content regions in the minor groove of the DNA helix, resulting in a DNA-dependent inhibition of RNA synthesis^54^. Mithramycin can also inhibit mRNA expression, thereby, resulting in reduced protein synthesis. In this study, mithramycin A was found to exhibit a dose-dependent inhibition of the toxin production by the bacteria when tested at subinhibitory concentrations of the drug. As represented in Fig. 1, mithramycin A exhibited approximately 9.7% and 27% inhibition in toxin production at 0.25X MIC and 0.5X MIC, respectively. Aureomycin, like other tetracyclines, is a bacterial protein synthesis inhibitor that prevents the interaction of the amino-acyl tRNA with the ribosome^39^. Indeed, aureomycin was found to inhibit toxin production by 15% and 50% at 0.25X MIC and 0.5X MIC, respectively. In fact, aureomycin was found to be more efficacious than fidaxomicin, which is one of the drugs of choice and is known to interfere with the toxin production by the pathogen^53^.

## Conclusion

The study involved screening the natural products set III library and the approved oncology drugs set V library in an attempt to identify potent inhibitors and drugs for the treatment of *C. difficile*. Of the 231 drugs/ compounds screened, 24 drugs/ compounds were found to exhibit anticlostridial activity. Three of these drugs, mitomycin C, mithramycin A, and aureomycin, were selected to further study their activity against the pathogen. These drugs exhibited potent activity when tested against a panel of clinically relevant *C. difficile* strains. In addition, two of the drugs, mithramycin A and aureomycin, were found to inhibit toxin production at subinhibitory concentrations. Aureomycin is a member of the tetracycline class of antibiotics, which is known to be less harmful to the gut microflora than other classes of antibiotics. Mithramycin A and aureomycin are also poorly absorbed from the intestine, a much-coveted pharmacokinetic property in drugs designed to treat *C. difficile* infection. This report suggests that mitomycin C, mithramycin A, and aureomycin scaffolds may warrant consideration when designing novel therapeutics for the treatment of CDI.

## Materials and Methods

### Bacterial strains & reagents

The *C. difficile* strains used in this study were obtained from the Biodefense and Emerging Infections Research Resources Repository (BEI Resources, Manassas, VA) and the American Type Culture Collection (ATCC, Manassas, VA) (Supplementary Table S1). The strains were cultured in brain heart infusion broth (BHIS; brain heart infusion medium from Becton, Dickinson and Company, Cockeysville, MD), supplemented with yeast extract (Fisher Scientific, Hampton, NH), L-cysteine (Alfa Aesar, Haverhill, MA), resazurin, vitamin K1, and hemin (Sigma-Aldrich, St. Louis, MO). Phosphate buffered saline (PBS) was purchased from Corning (Corning, NY).

### Compounds and libraries

The natural products set III consists of 117 compounds which were selected from the NCI open repository of 140,000 compounds (Natural Product Set III: NatProd_3_structures.sdf). The natural products set III was provided in 1 μl of glycerol at a concentration of 0.2 M in two 96-well polypropylene microtiter plates (plate 13120880 and 13120881). The approved oncology drugs set V and the natural products set III were kindly provided by the NCI (Bethesda, MD). The approved oncology drugs set V consists of 114 FDA-approved drugs intended to enable drug discovery (approved oncology drugs set V: ApprovedOncDrugs_5_structures.sdf). The approved oncology drugs set V was provided in two 96-well polypropylene microtiter plates (plate 4803 and 4804) at a volume of 20 microliters and concentration of 10 mM dissolved in 100% DMSO.

Mitomycin C, mithramycin A (Cayman Chemicals, Ann Arbor, MI) and aureomycin (chlortetracycline hydrochloride) (Sigma-Aldrich, St. Louis, MO) were selected from the list of the hit compounds and purchased separately to confirm the results. Vancomycin hydrochloride (Gold Biotechnology, Olivette, MO) and fidaxomicin (Cayman Chemicals) were used as positive controls.

### Screening assay

The two libraries were screened at a final concentration of 16 μM against *C. difficile* ATCC BAA 1870. Briefly, the bacterial strain was streaked on Bacto brain heart infusion (BHI) agar plates supplemented with resazurin, hemin, vitamin K1, and L-cysteine (BHIS) and incubated anaerobically at 37 °C for a period of 48 hours. A single bacterial colony was taken from the agar plate, suspended in sterile PBS, adjusted to the turbidity of 0.5 McFarland solution, and diluted in BHIS broth to attain a bacterial concentration of approximately 5 × 10^5^ CFU/ml. The compounds were dispensed to the 96-well plates and 100 μl of the BHIS broth containing bacteria was added to each well of the 96-well plate. The plates were then incubated anaerobically at 37 °C for 48 hours. The drugs/ compounds that were found to inhibit the bacterial growth were deemed “hits”. The drugs/ compounds that did not exhibit activity were excluded from the study.

### Activity of the selected drugs against a panel of *C. difficile* strains

To determin**e** the exact minimum inhibitory concentrations (MICs) of the hit drugs/compounds identified in the initial screening assay, the hit drugs/compounds were further tested against *C. difficile* ATCC BAA 1870 as per the guidelines provided by the Clinical and Laboratory Standards Institute (CLSI), with slight modification^55^. Briefly, the *C. difficile* strain was cultivated on BHIS agar plates and grown for 48 hours at 37 °C under anaerobic conditions. The active drugs were selected and added to achieve a final concentration of 32 μM in the first row of the 96-well plate. The bacterial suspension was prepared, adjusted to a turbidity of 0.5 McFarland solution, and added to BHIS broth to attain a bacterial concentration of 5 × 10^5^ CFU/ml. The BHIS broth containing bacteria was then added to the plate, serially diluted, and incubated anaerobically at 37 °C for 48 hours. The MIC was defined as the lowest concentration of the drugs that inhibited bacterial growth after the incubation period of 48 hours.

Based on the MIC data of the hit drugs/compounds, mitomycin C, mithramycin A, and aureomycin were further investigated; these three compounds were purchased and tested against a panel of 20 *C. difficile* clinical isolates (Supplementary Table S1) as per the CLSI guidelines and as described above.

### Toxin inhibition assay

Based on their proposed mechanism of action, mithramycin A^56^ and aureomycin^57^, were investigated to evaluate their effects on toxin production by *C. difficile* ATCC BAA 1870, with slight modifications^58^. Briefly, *C. difficile* strain ATCC BAA 1870 was cultured on BHIS agar plates and incubated anaerobically for 48 hours at 37 °C. A few colonies of the bacteria were taken from the agar plate and suspended in BHIS broth to achieve a bacterial concentration of approximately 10^6^ CFU/ml. An aliquot of 500 μl of this bacterial solution was added to each eppendorf tube containing subinhibitory concentrations (0.25X MIC and 0.5X MIC) of each drug to be tested along vancomycin and fidaxomicin as controls. The experimental set up was incubated anaerobically at 37 °C for approximately 12 hours. Following the incubation, a portion of each suspension was serially diluted in BHIS broth, plated on BHIS agar plates, and incubated anaerobically for approximately 24 hours to determine the bacterial count in each treatment. The remaining portion was centrifuged at 10,000 rpm for 5 minutes, the supernatant was collected, and the toxin concentration in the supernatant for each treatment was determined using an enzyme- linked immunosorbent assay (ELISA) (tgc BIOMICS, GmbH, Mainz, Germany) as per the manufacturer’s instructions. The OD_450_ was measured by a spectrophotometer using the BioTek Gen 5 Version 2.09 Data Analysis Software (Winooski, VT, USA).

## Supplementary information

Supplementary Information.
