# Supplementary material for: Screening of Natural Products and Approved Oncology Drug Libraries for Activity against Clostridioides difficile
Source: Sci Rep. 2020 Apr 6;10:5966. doi: 10.1038/s41598-020-63029-0 (PMC7136261; doi:10.1038/s41598-020-63029-0)
Supplement: Supplementary file 1 — Supplementary Information. [file 41598_2020_63029_MOESM1_ESM.pdf]

Screening of Natural Products and Approved Oncology Drug Libraries for Activity against *Clostridioides difficile*

Rusha Pal[1] and Mohamed N. Seleem[1,2*]

[1] Department of Comparative Pathobiology, College of Veterinary Medicine, Purdue University, West Lafayette, IN, 47907, USA
[2] Purdue Institute of Inflammation, Immunology, and Infectious Disease, West Lafayette, IN, 47907, USA

*Corresponding Author:
Mohamed N. Seleem
Department of Comparative Pathobiology
Purdue University College of Veterinary Medicine
625 Harrison St., West Lafayette, IN, 47907
Phone: 765-494-0763 Fax: 765-496-2627

Table S1: Bacterial strains used in the study.

Bacterial strains / ID number	Source and comments
P2 /NR-32883	Toxin producing strain isolated from patient stool in western Pennsylvania, USA in 2001
P6/ NR-32886	Toxin producing strain isolated from the stool of a patient suffering from recurrent *C. difficile* infection in western Pennsylvania, USA in 2001
P7/ NR-32887	Toxin producing strain procured from patient stool in western Pennsylvania, USA in 2001
P8/ NR-32888	Toxin producing strain procured from patient stool in western Pennsylvania, USA in 2001
P9/ NR-32889	Toxigenic strain procured from fecal matter of a patient suffering from recurrent *C. difficile* infection in western Pennsylvania, USA in 2001
P19/ NR-32895	Toxigenic strain procured from fecal matter of a patient suffering from recurrent *C. difficile* infection in western Pennsylvania, USA in 2005
Isolate 1/ NR-13427	Isolated from a patient diagnosed with CDI in the Mid-Atlantic region of the USA in 2008/2009
Isolate 2/ NR-13428	Isolated from a patient diagnosed with CDI in the Mid-Atlantic region of the USA in 2008/2009
Isolate 4/ NR-13430	Isolated from a patient diagnosed with CDI in the Mid-Atlantic region of the USA in 2008/2009
Isolate 6/ NR-13432	Isolated from a patient diagnosed with CDI in the Mid-Atlantic region of the USA in 2008/2009
Isolate 9/ NR-13435	Isolated from a patient diagnosed with CDI in the Mid-Atlantic region of the USA in 2008/2009
Isolate 1/ NR-13436	Isolated from a patient diagnosed with CDI in the Mid-Atlantic region of the USA in 2008/2009
Isolate 20100502/ NR-49277	Isolated in 2010 from the fecal matter of an elderly male patient diagnosed with community-associated (CA) *C. difficile* infection in Colorado, USA
Isolate 20100207/ NR-49278	Isolated in 2010 from the stool of an elderly adult male patient diagnosed with healthcare-associated (HA) *C. difficile* infection in New York, USA
Isolate 20100211/ NR-49279	Isolated in 2010 from the stool sample of a pediatric female patient diagnosed with community-associated (CA) *C. difficile* infection in New York, USA
Isolate 20120016/ NR-49282	Isolated in 2011 from the stool sample of a pediatric female patient diagnosed with community-associated (CA) *C. difficile* infection in New York, USA

Isolate 20110999/NR-49286	Isolated in 2011 from the stool sample of an elderly female patient diagnosed with healthcare-associated (HA) *C. difficile* infection in western/midwestern, USA
Isolate 20110870/ NR-49288	Isolated in 2011 from the stool sample of a young adult female patient diagnosed with healthcare associated (HA) *C. difficile* infection in Tennessee, USA
Isolate 20120187/ NR-49290	Isolated in 2011 from the stool sample of an elderly adult male patient with healthcare-associated (HA) *C. difficile* infection in Tennessee, USA
ATCC BAA 1870	Presence of *cdtB*[a], *tcdA*[b], and *tcdB*[c] genes, classified as toxinotype IIIB, ribotype 027

[a] cdtB= *C. difficile* binary toxin
[b] tcdA= *C. difficile* toxin A gene
[c] tcdB= *C. difficile* toxin B gene

Table S2: Initial screening data, chemical structure, and description of the hits for the approved oncology drugs set V library against *C. difficile* ATCC BAA 1807.

	Compound name	Chemical structure	MIC (µM)	Description and use
1	Ponatinib	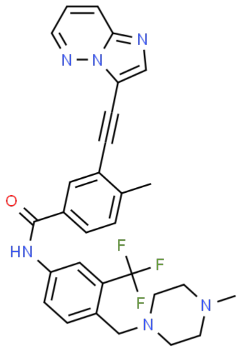	16	Tyrosine kinase receptor inhibitor that used in the therapy of refractory chronic myelogenous leukemia (CML).
2	Regorafenib	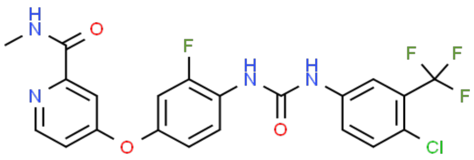	8	Oral multi-kinase inhibitor used in the therapy of refractory metastatic colorectal cancer, hepatocellular carcinoma and gastrointestinal stromal tumor.

3	Sorafenib	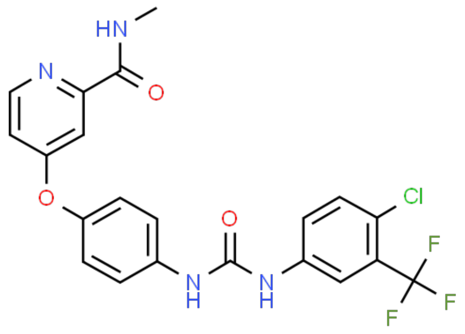	8	Protein kinase inhibitor used in the therapy of advanced renal cell, liver, and thyroid cancer.
4	Mitomycin C	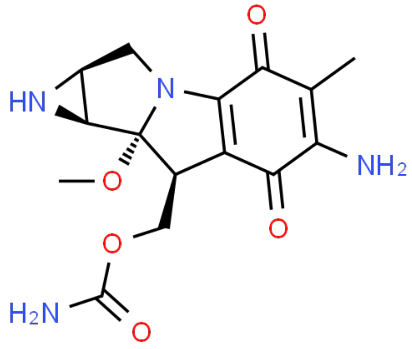	0.5	Antineoplastic antibiotic used in stomach and pancreatic cancers.
5	Tamoxifen citrate	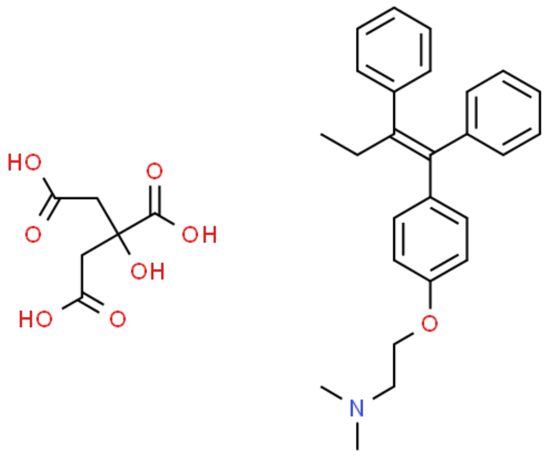	16	Selective estrogen receptor modulator used in malignant glioma and other cancers overexpressing protein kinase C expression.

6	Actinomycin D	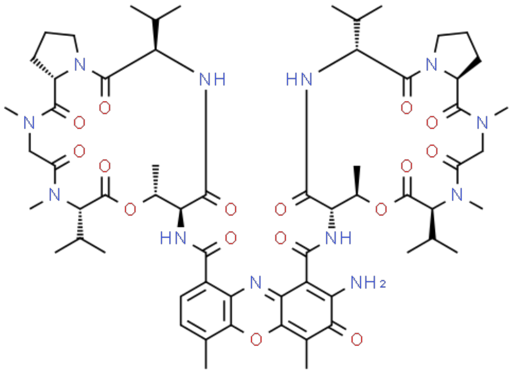	32	Antineoplastic antibiotic used in treating solid tumors in children and choriocarcinoma in adult women.
7	Mithramycin A/ Plicamycin	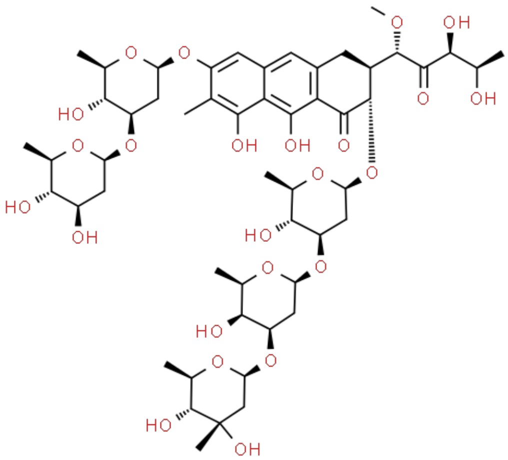	≤0.25	Antineoplastic antibiotic used in the treatment of testicular cancer.

Table S3: Initial screening data, chemical structure, and description of the hits for the natural product set III library against *C. difficile* ATCC BAA 1807:

	Compound name	Chemical structure	MIC (μM)	Description and use

1	Aureomycin	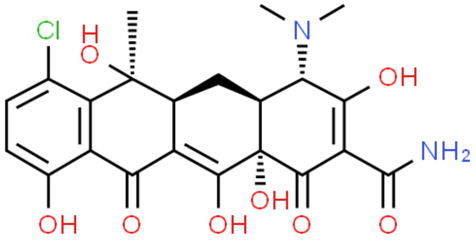	0.5	An antiprotozoal and antibacterial drug.
2	Norlobaric acid	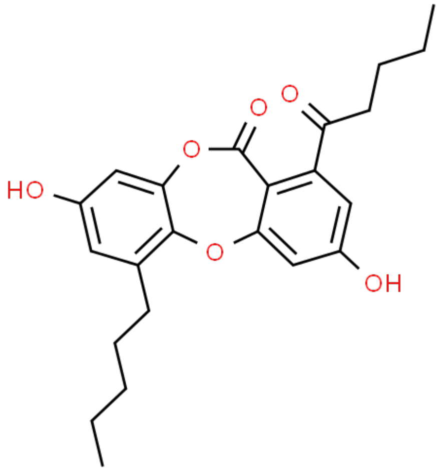	32	--
3	Actinomycin D	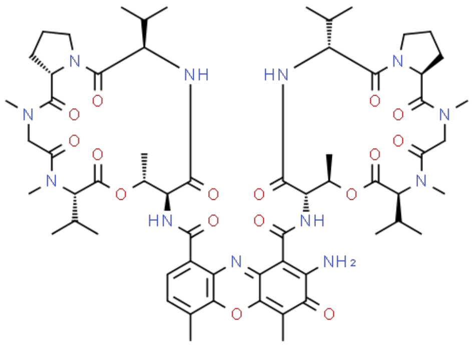	32	Antineoplastic antibiotic

4	Valinomycin	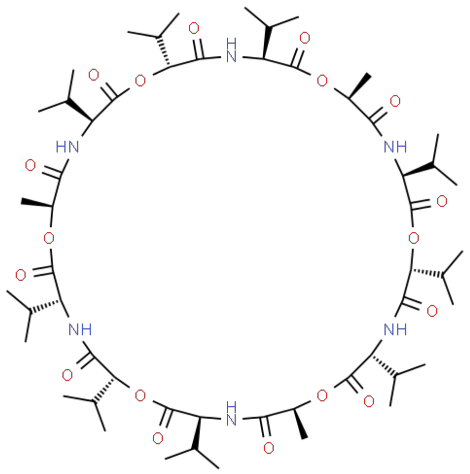	32	Antibiotic
5	Pomiferin	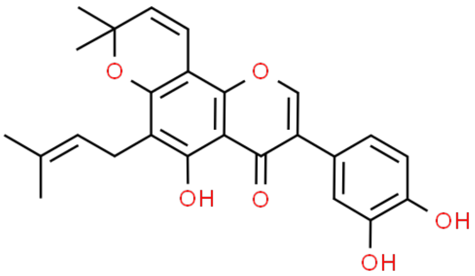	32	A prenylated isoflavone found in *Maclura pomifera*
6	Aristolochin	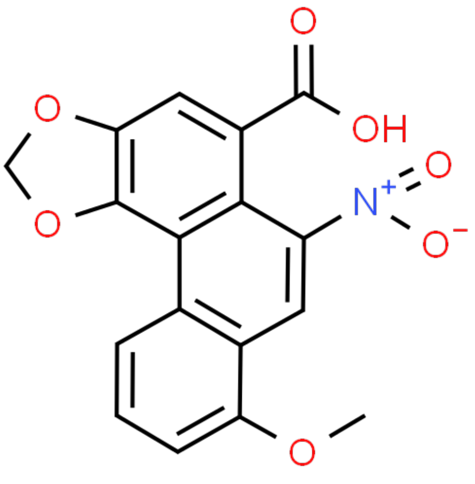	32	Monocarboxylic acid found in *Aristolochia* sp.

7	Mangostin	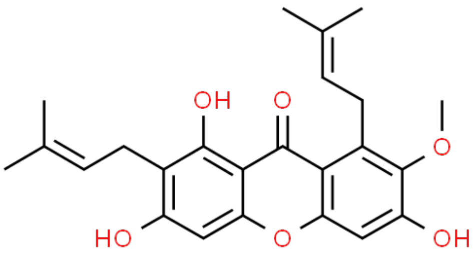	16	Antineoplastic antibiotic isolated from the stems of *Cratoxylum cochinchinense*,
8	Siomycin A	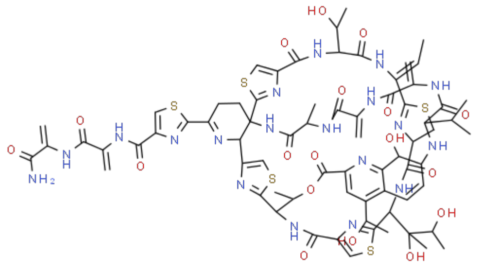	≤0.25	Antibiotic, antineoplastic
9	Lonchocarpic acid	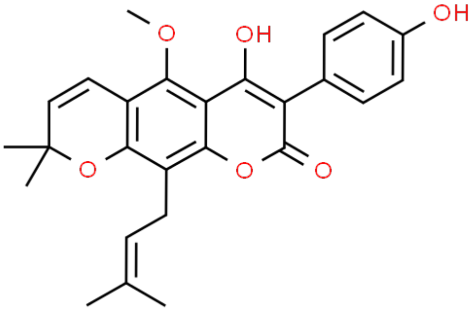	32	Isoflavonoid

10	Tetrocarcin A, sodium salt	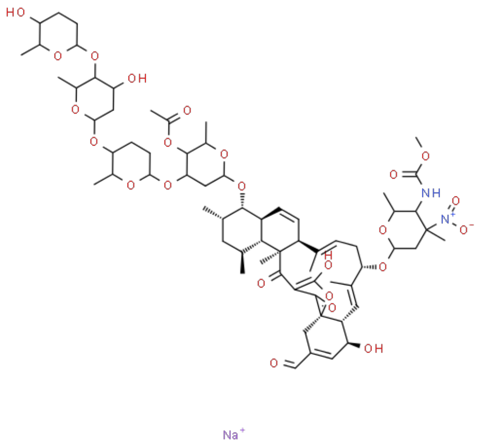	0.5	Antitumor antibiotics
11	Michellamine B	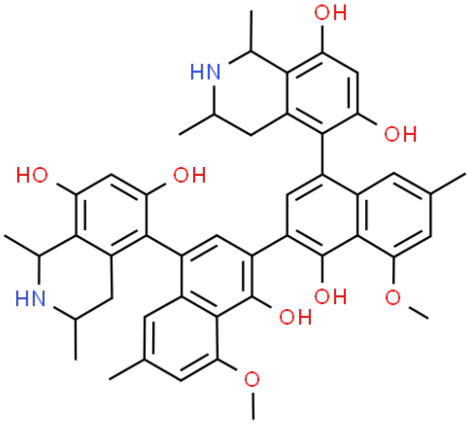	16	Novel plant alkaloid inhibiting HIV virus
12	Rifamycin	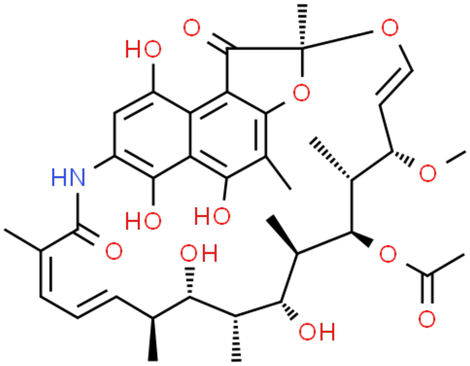	≤0.25	Natural antibiotic

13	Nigericin	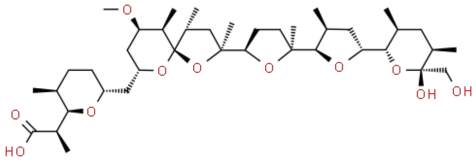	≤0.25	Polyether antibiotic
14	Antibiotic X-536A	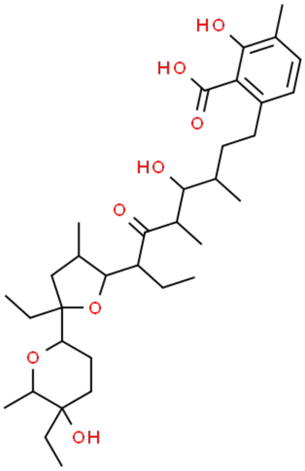	1	--
15	Chaetochromin	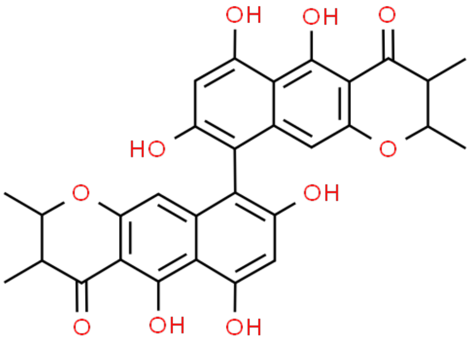	0.5	Small molecule obtained from *Chaetomium gracile* fungi.

16	Levomycin	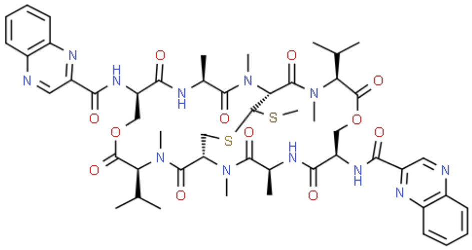	≤0.25	Polypeptide antibiotic
17	Gangetin	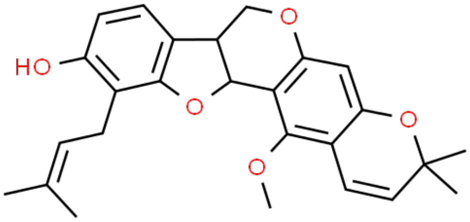	8	Hexane extract from the root of the plant *Desmodium gangeticum*
